# Self-assessment of oral hygiene in children aged 9‒14 years

**DOI:** 10.34172/japid.2023.008

**Published:** 2023-04-29

**Authors:** Ilma - Robo, Saimir Heta, Sonila Kapaj, Mario Llanaj, Vera Ostreni

**Affiliations:** ^1^Department of Therapy, Faculty of Dental Medicine, University of Medicine, Tirana, Albania; ^2^Pediatric Surgery, University Hospital, Tirana, Albania; ^3^Department of Gynecology, Hospital Center, Fier, Albania; ^4^Department of Dentistry, Faculty of Medical Sciences, Albanian University, Tirana, Albania; ^5^Department of Morphology, University of Medicine, Tirana, Albania

**Keywords:** Bacterial plaque, Hemorrhage index, Oral hygiene

## Abstract

**Background.:**

Self-assessment of oral hygiene can be well-accomplished through questionnaires with questions that can logically check deliberately erroneous answers by the individual being questioned.

**Methods.:**

The standard questionnaires were distributed to children aged 9‒14 years to collect information on the personal level of oral hygiene, find the reasons for not receiving correct information from the individuals and not referring to a dentist for routine visits, and determine fears arising from previous visits and procedures.

**Results.:**

Deliberately chosen incorrect answers comprised 6.3% of cases. Dental hygiene was expressed in the correlation of the hemorrhage index and the presence of bacteria in 72.4% of cases.

**Conclusion.:**

The dentist’s approach toward pediatric patients, especially young children, should encourage children to continue dental treatments and not postpone them due to pain and aggravated dental situations. The relatively small percentage of cases with high psychological stress during dental visits in this study was a positive aspect.

## Introduction

 Oral hygiene involves maintaining and controlling the amount of bacterial plaque deposited on the hard surfaces of teeth in the oral cavity. It is based on motor actions and “habits” that individuals have coordinated, learned, and acquired in time. Oral hygiene should begin when the first temporary tooth erupts in the oral cavity, performed under the supervision of the child’s parent. Then, everything depends on the individual, i.e., the growing child, to learn oral hygiene stages.^1-5^

 The instruments with which oral hygiene is performed are numerous, and the selection between different types of these instruments requires proper advice from a dentist, including tips on choosing a toothbrush, toothpaste, interdental floss, interdental brushes, mouthwashes, etc. The selection process does not rely only on individual taste but is based on the adaptation of these instruments to the oral cavity and specific individual requirements for achieving oral hygiene at an appropriate level.^2,6^

 The present study aimed to present data on oral hygiene and the approach of individuals toward solving oral problems concerning contact with a dentist and the frequency of dental treatments.^3,6-9^

 In addition to maintaining oral hygiene, part of dental care and the culture of maintaining oral hygiene is also part of dental control in dental clinics. Professional dental examinations and counseling by a dentist affect what the patient absorbs and perceives. The sensation of pain or trauma during dental intervention or treatment negatively affects the communication between the patient and the dentist. This part is dealt with in certain parts of the questionnaire.^1,9-11^

## Methods

 The study collected data related to oral hygiene from a cross-sectional perspective. Data were collected through questionnaires designed for this study on students aged 9‒14 in 2021. Due to the difficulty of collecting information during the COVID-19 pandemic, data were collected from 52 individuals. There is no repetition of the figures, but reprocessing and re-presentation of the data collected are other aspects.

 Collecting data from the group of children included in the study best fulfills the purpose of this study as possible correlations of individual importance to oral hygiene evolve from childhood to adolescence.

 This study was approved by the Institutional Ethics Committee of Albanian University (02.06.2021), Tirana, Albania, according to national regulations.

 After collecting the data in a basic table of excel, they were processed to display the study results according to the tables below.

## Results

 After collecting the study data in the basic Excel table, they were processed to display the results in Tables 1 to 6. Continuous data were presented in mean values and standard deviations, while discrete data were presented in absolute values and percentages. Graphs and tables of different types (simple and complex) were used to present the study’s data. Fisher’s exact test was used to make comparisons in the 2 × 2 contingency table of the collected data. *P* values < 0.05 were considered statistically significant.

**Table 1:** The individuals included in the study, classified based on the correct and incorrect answers on the checks and visits to the dentist, by gender

**Patients**	**Dental visits**							
	**Frequency** **(correct)**	**%**	**Frequency** **(incorrect)**	**%**	**Last visit** **(incorrect)**	**%**	**Last visit** **(correct)**	**%**
**Female**	9	17%	14	27%	4	8%	20	38%
**Male**	7	13%	22	42%	5	10%	23	44%
**Total**	16	31%	36	69%	9	17%	43	83%

**Table 2:** Patients divided based on the sensation, reasons, and experience of the first visit to a dentist

**The first visit - perception sense**	**Pain**	**I did not feel comfortable**	**I felt nothing**	**The treatment was performed**	**Total (%)**
Terrified	6	-	-	-	6‒12%
Scared	5	3	-	-	8‒15%
A little scared	13	3	2	-	18‒35%
There was no fear at all	11	3	5	1	20‒38%
Total (%)	35‒67%	9‒17%	7‒13%	1‒2%	52‒100%

**Table 3:** Patients classified on the basis of experience and the reason of the last visit

**Last dental visit**	**Toothache**	**Parent / friend advice**	**Dentist reference**	**Other reasons**	**Total-%**
≤ 6 months	19	3	-	13	35-67%
6-12 months	5	2	-	1	8-15%
1-2 last years	4	1	-	-	5-10%
2-5 last years	3	-	-	1	4-8%
Total-%	31-60%	6-12%	0-0%	15-29%	52-100%

**Table 4:** Bacterial plaque and gingival bleeding seen from the perspective of individuals with the answers to questions 7‒10 of the questionnaire

**Bleeding index Bacterial plaque**	**0-1**	**2**	**3**	**4**	**5**	**Total - %**
Soft mass	3	1	9	-	1	14‒13%
Strong mass	1	-	12	-	2	15‒14%
Fixed mass	2	-	8	-	2	12‒12%
Causes swelling	-	-	9	-	1	10‒10%
Tooth coloring	2	2	8	2	3	17‒16%
Caries	2	-	5	-	2	9‒9%
I do not know anything	12	-	12	-	3	27‒26%
Total (%)	22‒21%	3‒3%	63‒61%	2‒2%	14‒14%	104-100%

**Table 5:** Dental brushing technique and instruments used for oral hygiene, seen from the perspective of individuals with the answers to questions 11‒16 of the questionnaire

**Instruments**	**Hygiene technique**			
	Correct**	**Partly correct**	I**ncorrect**	**Total (%)**
Correct*	6	10	2	18‒35%
Partial correct	1	12	12	25‒48%
Incorrect	2	4	3	9‒17%
Total (%)	9‒17%	26‒50%	17‒32%	104‒100%

*The hygienic technique was correct. The rinsing of the oral cavity was performed twice a day (in the morning and after dinner), the duration of one wash was at least 3 minutes, and the rinsing was carried out before urination. **Instruments used included brush, paste, dental floss, and mouthwash. The brush was changed once in 6 months. Brushing was carried out with mechanical cleaning movements without any preference for any movement.

**Table 6:** Caries from the perspective of individuals with the answers to questions 17‒21 of the questionnaire

**Caries-dental restoration**					
**Influencing factors**	**Caries**		**Restored teeth**		**Total (%)**
	**Yes**	**No**	**Yes**	**No**	
Diets	17	26	29	14	86‒55%
	3	5	7	4	19‒12%%
Carious teeth in appearance	11	17	19	9	56‒36%
	9	16	14	9	48‒31%
Prevention	17	28	31	14	90‒58%
	2	5	2	4	13‒8%
Total (%)	59‒38%	97‒63%	102‒65%	54‒35%	312‒100%

**Figure 1:**
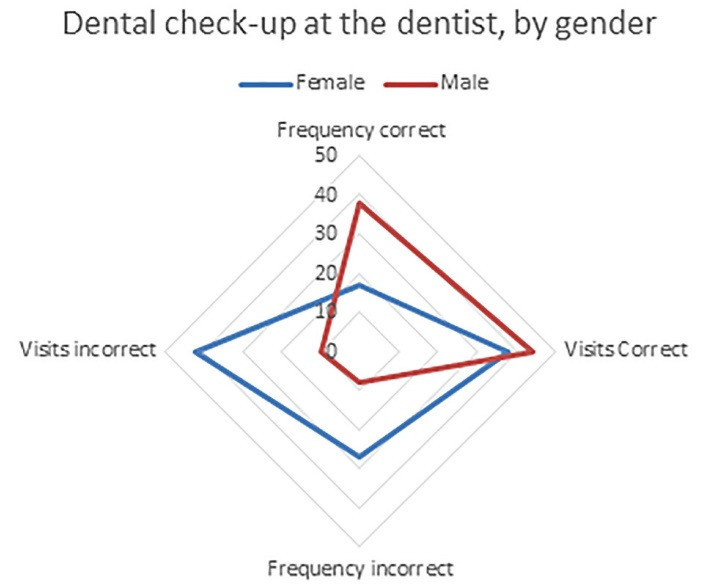
The graph presents the data of Table 1, with approximate percentages of healthy and not healthy patients

**Figure 2:**
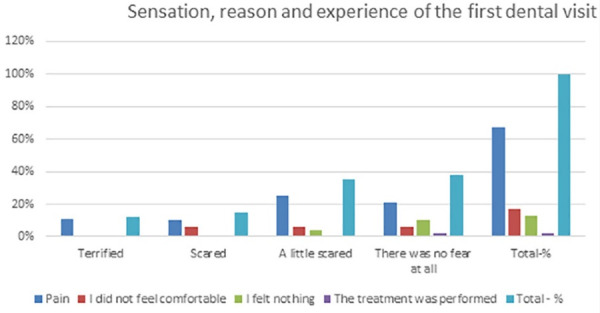
Patients categorized based on the sensation, reasons, and experience of the first dental visit

**Figure 3:**
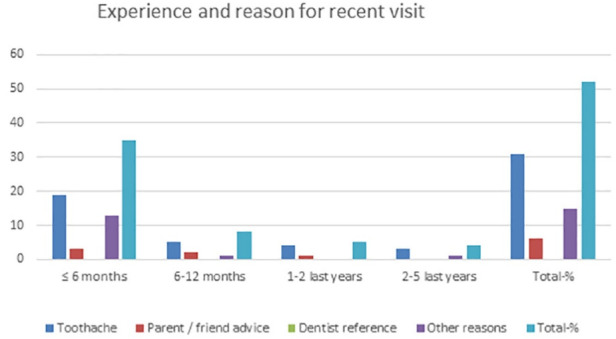
Patients classified based on experience and the reason for the last dental visit

**Figure 4:**
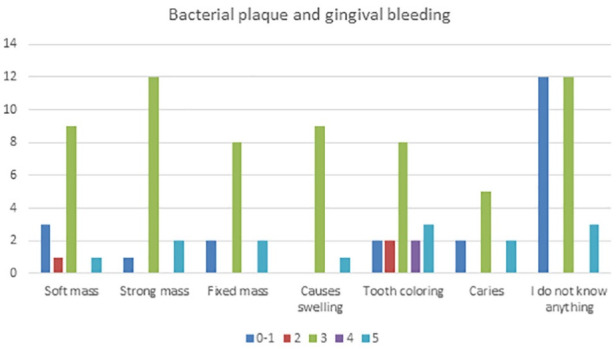
Bacterial plaque and gingival bleeding seen from the perspective of individuals with the answers to questions 7‒10 of the questionnaire

 The collected data shown in Table 1 were classified based on the correct or incorrect answers about proper control and dental visits, divided by the gender of students aged 9‒14. The data from Table 1 are presented graphically in Figure 1. This graph presents the data of Table 1, with approximate percentages of healthy and not healthy patients. Patients divided based on the sensation, reasons, and experience of the first visit to a dentist are the data presented in Table 2 and graphically in Figure 2. Patients classified based on experience and the reason for the last dental visit are presented in the data in Table 3 and graphically in Figure 3. Table 3 and the graph in Figure 4 show data on bacterial plaque and gingival bleeding seen from the perspective of individuals with the answers to questions 7‒10 of the questionnaire.

**Figure 5:**
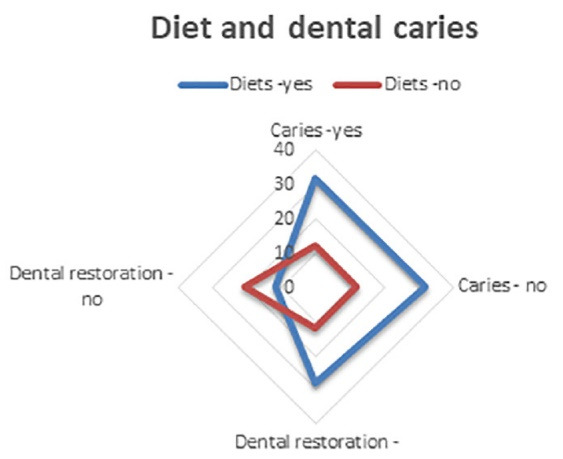
Status of patients’ care-dental restoration and influencing factors before looking at individuals with the answers to questions 17‒21 of the questionnaire concerning the impact of diet

**Figure 6:**
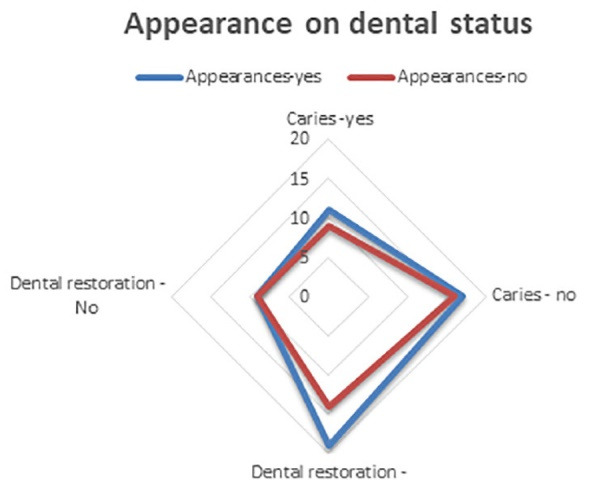
Status of patients’ care-dental restoration and influencing factors from the perspective of individuals with the answers to questions 17‒21 of the questionnaire concerning the impact of appearance

**Figure 7:**
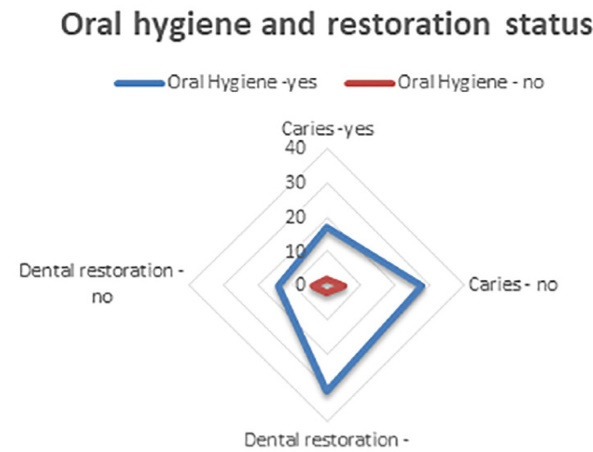
Status of patients’ care-dental restoration and influencing factors from the perspective of individuals with the answers to questions 17‒21 of the questionnaire concerning the impact of prevention on oral hygiene

 Dental brushing technique and instruments used for oral hygiene, seen from the perspective of individuals with the answers to questions 11‒16 of the questionnaire, are data presented in Table 4, while Table 5 presents data about caries from the perspective of individuals with the answers to questions 17‒21 of the questionnaire. Figure 5 graphically presents the collected data about the status of patients’ care and dental restorations and influencing factors before looking at individuals, with the answers to questions 17‒21 of the questionnaire concerning the impact of diet. Figure 6 graphically presents the data about the status of patients’ care and dental restorations and influencing factors from the perspective of individuals, with the answers to questions 17‒21 of the questionnaire concerning the impact of appearance. Figure 7 presents data about the status of patients’ care and dental restorations and influencing factors from the perspective of individuals, with the answers to questions 17‒21 of the questionnaire concerning the impact of prevention on oral hygiene.

## Discussion

 Identifying the performance of the proper oral hygiene techniques is carried out by bacterial staining in the dental clinic. These oral dyes can stain the remaining bacterial plaque to identify it in areas where bacteria have not been removed. Removal of bacteria is a mechanical action, as the bond between the tooth and bacteria is weak and can be disrupted by mechanical forces between the brush and toothpaste and the tooth surface without causing the loss of tooth structure.^3,6,8,11^

 Oral hygiene is the main element in preventing and treating periodontal diseases. Oral hygiene maintenance and methods to achieve proper oral hygiene are two elements that should be included in the individual’s education. The initial stages of teaching and practicing oral hygiene coincide from a young age. These stages should be accompanied by additional information passed from parents to children to achieve the child’s perception concerning the role of proper oral hygiene in the child’s current oral health and then the adult individual after a few years.^1,5,7,10^

 Evaluation of the pain sensation during the first dental visit showed a positive rate of 67%, and only 2% of patients realized that dental treatment had been performed. A feeling of pain associated with a feeling of psychological stress reached 12% of cases. Psychological stress affects dental emergencies caused by dental interventions, but subjectively this sensation reached 62%.

 The reason for the last visit to the dentist was pain in 60% of cases, parent/friend counseling in 12%, and referrals to dentists in 0%. Divided by the period completed: < 6 months: 67%, at an interval of 6‒12 months: 15%, in the last 1‒2 last years: 10%, and in the last 2‒5 years: 8%.

 Patients’ perception of bacterial aggregation as a soft mass was 13%, a strong mass and visual confusion with periodontal stone was 14%, and a fixed mass was 12%. The sensation and logic that bacterial plaque causes diseases was 10% for periodontal diseases and 9% for the appearance of caries.

 Approximately 83% of students aged 9‒14 knew how to brush their teeth well, with only 35% using proper methods and tools for brushing; 17% of students did not know how to wash the oral cavity.

 55% of students knew that a wrong diet with the wrong ingredients caused caries because they had carious teeth in their oral cavities, and 15% were caries-free.

 The impact of restorations and carious teeth was reflected in the appearance and self-esteem of this age group: % and 36%, respectively.

 Prevention of caries and periodontal diseases through oral hygiene was carried out in 58% of cases.

## Conclusion

 Dentists’ approach toward pediatric patients should positively impact children, encouraging them to continue dental treatments. Emphasis should be placed on dental prophylaxis. Periodontal health assessment should be performed according to the combination assessment with the index of bacterial places and index of gingival bleeding, as the correlation of these two indexes is more functional and easily recordable for this age.

## Acknowledgments

 None.

## Availability of Data

 The datasets analyzed during the current study are available from the corresponding author.

## Competing Interests

 The authors declare that they have no competing interests.

## Ethical Approval

 This study was approved by the Albanian University Institutional Ethics Committee (02.06.2021), Tirana, Albania, according to national regulations.

## Funding

 No funding for this research.

## References

[1] Priya M, Devdas K, Amarlal D, Venkatachalapathy A (2013). Oral health attitudes, knowledge and practice among school children in Chennai, India. J Educ Ethics Dent.

[2] Grossi SG, Zambon JJ, Ho AW, Koch G, Dunford RG, Machtei EE (1994). Assessment of risk for periodontal disease I. Risk indicators for attachment loss. J Periodontol.

[3] Tadjoedin FM, Fitri AH, Kuswandani SO, Sulijaya B, Soeroso Y (2017). The correlation between age and periodontal diseases. J Int Dent Med Res.

[4] Robo I, Heta S, Lasku G, Ostreni V (2021). Gingival recession and attachment loss: cross-sectional and retrospective data of 10 years. J Adv Periodontol Implant Dent.

[5] Genco RJ (1996). Current view of risk factors for periodontal diseases. J Periodontol.

[6] Robo I, Heta S, Hamzai F, Ostreni V (2019). The effect of conservative periodontal therapy at patients with systemic diseases. Arch Intern Med Res.

[7] Kornman KS, Crane A, Wang HY, di Giovine FS, Newman MG, Pirk FW (1997). The interleukin-1 genotype as a severity factor in adult periodontal disease. J Clin Periodontol.

[8] Guthmiller JM, Novak KF. Periodontal diseases. In: Brogden KA, Guthmiller JM, eds. Polymicrobial Diseases. Washington, DC: ASM Press; 2002. 21735561

[9] Marsh PD (2006). Dental plaque as a biofilm and a microbial community - implications for health and disease. BMC Oral Health.

[10] Obregón-Rodríguez N, Fernández-Riveiro P, Piñeiro-Lamas M, Smyth-Chamosa E, Montes-Martínez A, Suárez-Cunqueiro MM (2019). Prevalence and caries-related risk factors in schoolchildren of 12- and 15-year-old: a cross-sectional study. BMC Oral Health.

[11] Kamran S, Moradian H, Yazdan Bakhsh E (2019). Comparison of the mean DMF index in type I diabetic and healthy children. J Dent (Shiraz).

